# Stratum Corneum Ceramide Abnormalities in Atopic Dermatitis: Pathophysiology and Implications for Disease Management

**DOI:** 10.1111/1346-8138.70098

**Published:** 2025-12-15

**Authors:** Takashi Sakai

**Affiliations:** ^1^ Department of Dermatology, Faculty of Medicine Oita University Yufu‐shi Oita Japan

**Keywords:** atopic dermatitis, ceramides, disease modification, lipids, skin barrier, stratum corneum

## Abstract

The stratum corneum, as the outermost layer of the skin, functions as a critical barrier that maintains cutaneous hydration and systemic homeostasis. Among its structural lipids, ceramides constitute the most abundant and diverse component. These molecules are essential for the formation of lamellar structures that secure barrier integrity. Increasing evidence has established that abnormalities in stratum corneum ceramides are not merely epiphenomena but fundamental contributors to the pathophysiology of atopic dermatitis (AD). In this review, we provide an overview of the structure, biosynthesis, and diversity of ceramides within the stratum corneum, followed by a discussion of their pivotal role in skin barrier function. We highlight recent insights into how ceramide abnormalities manifest in AD, including reduced total content, altered class distribution, and a shift toward shorter‐chain fatty acids. Such alterations are associated with increased transepidermal water loss and impaired hydration. Mechanistic studies further reveal that type 2 cytokines, particularly IL‐4 and IL‐13, directly disrupt lipid metabolism by inhibiting enzymes, thereby establishing a vicious cycle of inflammation and barrier dysfunction. Beyond pathophysiology, advances in lipidomics and tape‐stripping techniques now enable noninvasive assessment of stratum corneum ceramides. These analyses have revealed their utility as biomarkers of disease activity, therapeutic response, and relapse risk. Collectively, ceramides of the stratum corneum provide a unique window into the biology of AD. Their accessibility, mechanistic relevance, and prognostic potential underscore their importance not only for understanding disease pathogenesis but also for advancing personalized management and the concept of disease modification in AD.

## 1 Structure and Diversity of Stratum Corneum Ceramides: Insights from Recent Lipidomics Studies

The skin constitutes the largest organ of the human body, covering the entire external surface and forming a dynamic interface between the internal milieu and the external environment. It is not merely a passive covering but rather serves as an essential barrier organ, one that plays a decisive role in maintaining systemic homeostasis and in safeguarding life itself. The architectural integrity of the skin barrier, encompassing both its macroscopic structure and its intricate cellular and molecular composition, is therefore of paramount physiological significance. Disruption of this barrier readily precipitates the onset and progression of numerous cutaneous as well as systemic disorders. Within this highly specialized barrier, ceramides of the stratum corneum occupy a central position, playing a pivotal role in maintaining epidermal integrity and barrier function. In this review, written from a dermatology‐oriented clinical perspective, we highlight recent advances in the understanding of stratum corneum ceramides—particularly their structural diversity revealed by lipidomics, their emerging connection with type 2 inflammation, and their potential clinical applications as biomarkers in atopic dermatitis (AD) [1, 2, 3, 4, 5].

**FIGURE 1:**
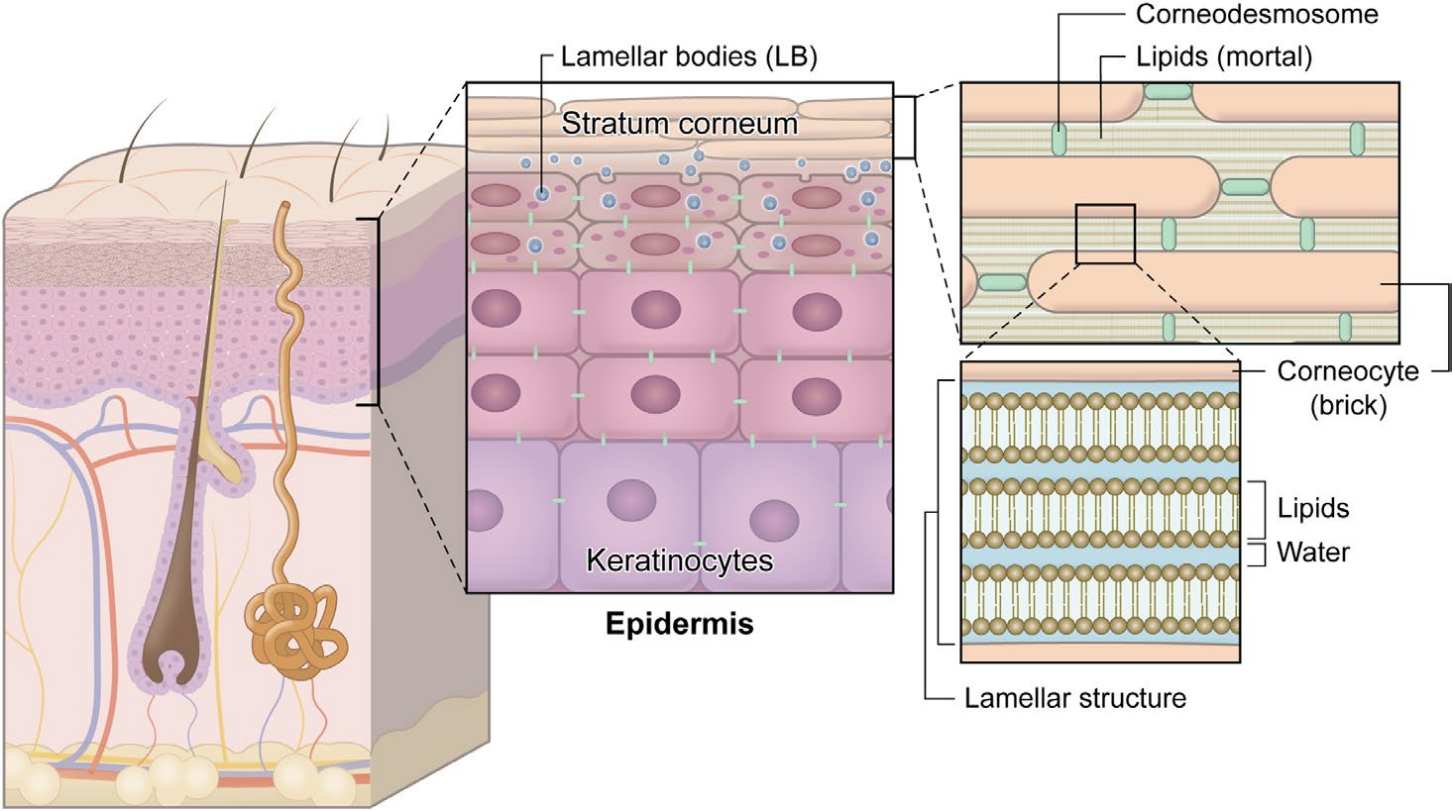
Stratum corneum lipids and lamellar architecture. Keratinocytes proliferate in the basal layer and migrate through the prickle and granular layers to form the stratum corneum, which consists of approximately 10 layers of flattened, anucleate corneocytes that are ultimately shed as squames. In the granular layer, lamellar bodies (LBs) are formed and packed with lipid precursors—cholesterol sulfate, phospholipids, and glucosylceramide/sphingomyelin—together with enzymes required for their processing. As keratinocytes transition from the granular layer to the stratum corneum, the lipid contents of LBs are secreted into the extracellular space. These secreted lipids subsequently organize into lamellar structures, in which lipid and water layers are alternately stacked in a highly regular fashion.

The epidermis, which represents the outermost layer of the skin, is composed of a stratified squamous epithelium with an average thickness of approximately 0.2 mm. Among the epidermal cell populations, keratinocytes are predominant, accounting for approximately 95% of all epidermal cells. These keratinocytes proliferate within the basal cell layer and, as they differentiate and mature, migrate upward through the spinous (prickle) cell layer and the granular cell layer before ultimately forming the stratum corneum. The stratum corneum itself consists of roughly 10 layers of keratinocytes that have undergone terminal differentiation, lost their nuclei, and transformed into flattened corneocytes. These corneocytes are ultimately desquamated from the skin surface as squames [2]. Within the granular cell layer, specialized organelles termed lamellar bodies (LBs) are readily observed, and they serve as critical sites for the generation of stratum corneum lipids. The three principal classes of lipids—cholesterol, free fatty acids, and ceramides—are produced from cholesterol sulfate, phospholipids, and glucosylceramide/sphingomyelin. These lipid precursors are synthesized and packaged into LBs, and as keratinocytes migrate from the granular layer to the stratum corneum, the contents of LBs are secreted into the extracellular space. This secretion event initiates the assembly of the characteristic lamellar structures of the stratum corneum lipids. This highly ordered arrangement, in which lipids are intercalated between layers of corneocytes, has long been conceptualized as the “brick and mortar” model. Within this architectural paradigm, the corneocytes function as the “bricks,” while the lipid lamellae constitute the “mortar” that fills the intercellular spaces. The lipid lamellae themselves are organized into multilamellar arrays, consisting of alternating layers of lipid bilayers and associated water molecules. This lamellar arrangement is a key structural prerequisite for the barrier properties of the skin (Figure 1) [6].

**FIGURE 2:**
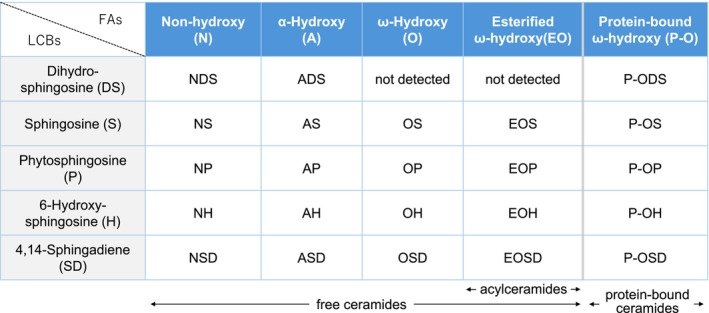
Twenty‐three ceramide classes identified in human stratum corneum. The prototypical ceramide molecule consists of a sphingoid long‐chain base (LCB) linked via an amide bond to a fatty acid (FA). Based on the combinations of five types of LCBs and five types of FAs, ceramides can be classified into 25 classes, of which 23 have been detected in the human stratum corneum. Each class is designated according to the specific pairing of an FA with an LCB, resulting in nomenclature such as NDS, NS, and NP. This figure was prepared based on the data reported in Ref. [10].

Importantly, the relative abundance of these lipid classes is maintained in approximate equimolar ratios, with ceramides representing the dominant component by weight, accounting for nearly half of the total lipid content. The biosynthesis of stratum corneum ceramides requires the coordinated action of multiple enzymes, including serine palmitoyltransferase, β‐glucocerebrosidase, sphingomyelinase, elongation of very long‐chain fatty acid (ELOVL) enzymes, and ceramide synthases. This finely balanced composition is indispensable for the proper formation of lipid lamellae and for ensuring the permeability barrier of the skin [6, 7]. Although ceramides are ubiquitously present in all tissues and cell types, they usually exist in only small amounts. By contrast, the stratum corneum is enriched with ceramides to a level that is several dozen times higher than that found in other tissues, and this lipid pool displays extraordinary structural diversity. The prototypical ceramide molecule consists of a sphingoid long‐chain base linked via an amide bond to a fatty acid, thereby producing a dihydrophobic structure. In the stratum corneum; however, unique subclasses are found, including acylceramides that incorporate linoleic acid to generate a trihydrophobic chain structure, and protein‐bound ceramides that covalently attach to proteins at the corneocyte surface, forming the corneocyte lipid envelope [6, 8, 9, 10]. Ceramides in the stratum corneum can be studied through tape‐stripping techniques followed by organic solvent extraction of lipids and subsequent chemical analyses. Masukawa and colleagues reported the presence of 11 distinct ceramide classes encompassing 342 individual molecular species within the human stratum corneum [11]. Classification of ceramides is based primarily on the type of sphingoid base and fatty acid moieties, and further subdivisions are determined by the number of total carbons, the degree and location of unsaturation, and other structural features. With the rapid evolution of lipidomic technologies—particularly the integration of liquid chromatography (LC) and mass spectrometry (MS)—comprehensive characterization of lipid species has become possible. Employing LC coupled with tandem mass spectrometry (LC–MS/MS), Suzuki et al. demonstrated the existence of 23 ceramide classes (Figure 2) encompassing 1581 molecular species, thus elucidating the full ceramide repertoire of the human stratum corneum [10]. The remarkable structural diversity of ceramides is not a mere biochemical curiosity; rather, it underpins the maintenance of skin barrier homeostasis and provides critical insights into dermatological disease, particularly in the context of AD [1].

## 2 Stratum Corneum Ceramides and Skin Barrier Function: Disruption by Environmental and Genetic Factors

The hydrophobicity of stratum corneum lipids enables them to retain water within the epidermis, and they therefore play a fundamental role in maintaining cutaneous hydration and barrier function. Experimental depletion of stratum corneum lipids, for instance by topical acetone treatment, leads to a marked increase in transepidermal water loss (TEWL) and a concomitant reduction in stratum corneum hydration. Both parameters are widely regarded as reliable indicators of skin barrier. Following acetone‐induced depletion, stratum corneum lipids are gradually replenished over approximately two days, during which TEWL and hydration levels also normalize [12, 13]. By contrast, when acetone‐treated skin is subsequently occluded with an impermeable membrane, lipid replenishment is markedly inhibited, demonstrating that barrier perturbation itself provides the stimulus for compensatory lipid synthesis [12]. Furthermore, acetone‐treated skin exhibits elongation of intraepidermal nerve fibers and an associated increase in scratching behavior [13, 14]. Importantly, analogous barrier disruption models have been established in human subjects as well, thereby confirming the translational significance of these findings [15].

In addition to acquired disruption, genetic defects in lipid metabolism also result in impaired barrier function. In Gaucher disease type 2, for example, deficiency or severely diminished activity of glucocerebrosidase abolishes ceramide generation in the stratum corneum, leading to ichthyosiform skin changes and barrier dysfunction [16]. Similarly, in Niemann–Pick disease characterized by profound acid sphingomyelinase deficiency, barrier recovery following tape‐stripping is markedly delayed [17]. Mutations in genes involved in the synthesis of acylceramides or protein‐bound ceramides have also been identified as causative in congenital ichthyoses [4, 18]. For example, mutations in ELOVL4, which is involved in the synthesis of acylceramides, cause ichthyosis symptoms with severe barrier defects in both human patients and mouse models [19, 20]. In addition, mutations in ALOX12B and ALOXE3, which participate in the formation of protein‐bound ceramides, are known to cause autosomal recessive congenital ichthyosis, and mice lacking these genes also exhibit marked skin barrier abnormalities [21, 22, 23]. Collectively, these clinical and experimental data firmly establish stratum corneum ceramides as indispensable components for maintaining barrier integrity and cutaneous homeostasis.

## 3 Stratum Corneum Ceramide Abnormalities in Patients With Atopic Dermatitis: Relationship Between Type 2 Inflammation and Lipid Alterations

AD is a chronic inflammatory skin disorder characterized by persistent barrier dysfunction, type 2 immune activation, and recurrent eczematous lesions associated with intense pruritus [24]. Classically, AD is linked with other atopic comorbidities such as food allergy, bronchial asthma, and allergic rhinitis [24]. More recently, however, attention has also been directed toward systemic complications of AD, including osteoporotic fractures [25, 26] and increased cardiovascular risk [27]. Altered composition and structural properties of stratum corneum ceramides are well recognized in AD [1, 2, 6]. Lesional skin in AD exhibits significantly decreased total ceramide content as well as reductions in specific classes such as NH, NP, EOS, EOH, and EOP, compared with normal skin [28] (see Figure 2 for classification). Moreover, ceramide profiles in AD are skewed toward shorter chain lengths, resulting in an increased prevalence of short‐chain ceramides within specific ceramide classes. These alterations correlate with elevated TEWL and reduced hydration, establishing a functional link between lipid abnormalities and barrier impairment [28, 29]. Mechanistically, inflammation plays a principal role in driving these abnormalities [29].

In both patients with atopic dermatitis and IL‐13 skin‐specific transgenic mice, analysis of stratum corneum lipids has demonstrated a significant shift in lipid composition compared with healthy controls. Specifically, there is an increased proportion of short‐chain NS ceramides, sphingomyelins, and C14:0–C22:0 lysophosphatidylcholines, accompanied by a corresponding reduction in the levels of their long‐chain counterparts. Furthermore, IL‐4 and IL‐13 have been shown to suppress the expression of ELOVL enzymes 3 and 6 in cultured keratinocytes through a signal transducer and activator of transcription 6 (STAT6)‐dependent mechanism [30]. Additional evidence from experiments using human epidermal sheets further supports this mechanistic link: tumor necrosis factor‐α and interferon‐γ enhance ceramide synthesis and upregulate the activities of sphingomyelinase and glucocerebrosidase, both of which catalyze critical steps in ceramide generation. By contrast, IL‐4 effectively inhibits these processes [31]. The association between type 2 inflammation, stratum corneum ceramides, and ceramide‐related enzymes observed in human studies is summarized in Table 1 [28, 30, 31, 32]. Collectively, these findings strongly suggest that type 2 inflammatory cytokines directly perturb stratum corneum lipid metabolism and thereby contribute to barrier dysfunction in AD.

**TABLE 1:** Association between type 2 inflammation, stratum corneum ceramides, and ceramide‐related enzymes in human samples.

Evaluation method	Effects of type 2 inflammation on ceramide‐related enzymes or resulting stratum corneum ceramide abnormalities	References
AD lesional skin	Decreased total ceramide content and reduced levels of several classes (NH, NP, EOS, EOH, EOP), and a shift toward shorter‐chain species in some ceramide classes compared with normal skin.	[28]
AD lesional skin	Expression levels of ELOVL3 and ELOVL6 were decreased in AD lesional skin compared with healthy controls.	[30]
Human keratinocytes	Stimulation with IL‐4 and IL‐13 reduced the expression of ELOVL3 and ELOVL6 in cultured keratinocytes.	[30]
Human epidermal sheets	TNF‐α and IFN‐γ enhance ceramide synthesis by upregulating sphingomyelinase and glucocerebrosidase, whereas IL‐4 inhibits these enzymes.	[31]
AD lesional skin	Sphingomyelinase activity is reduced in AD lesional skin compared with healthy controls.	[32]

Abbreviations: AD, atopic dermatitis; ELOVL, elongation of very long‐chain fatty acids; IFN, interferon; IL, interleukin; SC, stratum corneum; TNF, tumor necrosis factor. For detailed classification of ceramide subclasses (NH, NP, EOS, EOH, and EOP), please see Figure 2.

**FIGURE 3:**
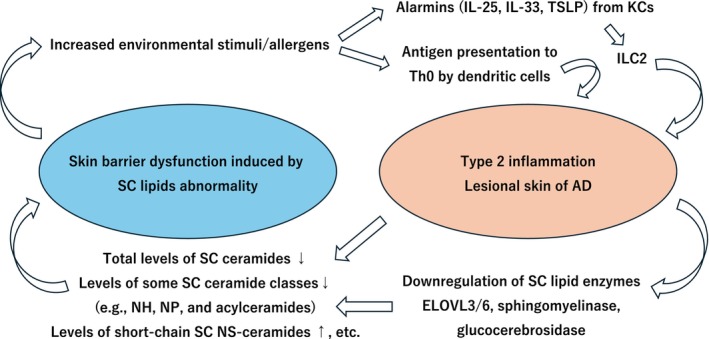
Vicious cycle between type 2 inflammation and stratum corneum lipid abnormalities (skin barrier dysfunction). In atopic dermatitis, a disease characterized by type 2 inflammation, cytokines such as IL‐4 and IL‐13 contribute to disease pathogenesis. These type 2 cytokines suppress stratum corneum (SC) lipid–synthesizing enzymes, including elongation of very long‐chain fatty acid (ELOVL) enzymes 3 and 6, sphingomyelinase, and glucocerebrosidase. As a result, the total levels of ceramides in the stratum corneum, as well as the levels of certain ceramide classes such as NH, NP, and acylceramides, decrease, whereas the levels of short‐chain NS ceramides increase. These lipid abnormalities lead to skin barrier dysfunction, and once the barrier is weakened, penetration of external stimuli and allergens is facilitated. This triggers the production of epithelial‐derived alarmins from keratinocytes (KCs), followed by the activation of group 2 innate lymphoid cells (ILC2s) or antigen presentation by dendritic cells to Th0 cells. Both pathways further amplify type 2 inflammation, thereby establishing a self‐perpetuating vicious cycle.

Notably, disruption of the stratum corneum barrier not only weakens the structural integrity of the skin but also facilitates the entry of exogenous irritants and allergens. This enhanced permeability promotes antigen presentation by dendritic cells, drives type 2 immune responses, and induces the production of epithelial‐derived alarmins such as interleukin (IL)‐25, IL‐33, and thymic stromal lymphopoietin (TSLP). These alarmins further activate group 2 innate lymphoid cells (ILC2s), thereby amplifying type 2 inflammation. As a result, a self‐perpetuating vicious cycle of barrier disruption and type 2 immune activation is established in the pathophysiology of atopic dermatitis (Figure 3) [33].

When evaluating stratum corneum ceramides, it is essential to recognize that multiple factors may influence their levels and composition. These include anatomical differences across body sites, environmental influences, systemic diseases, as well as the effects of inflammation and genetic background, as discussed above. For instance, the relative abundance of ceramides has been reported to be higher in the wrist compared with the upper forearm [34], and adaptive regulation of stratum corneum lipids in response to temperature has also been demonstrated [35]. Indeed, seasonal and regional variations in ceramide profiles have been documented, with measurable differences depending on both the site of sampling and the time of year [36, 37, 38]. Moreover, patients with type 2 diabetes mellitus exhibit reduced stratum corneum hydration and lower ceramide content compared with healthy controls [39]. Importantly, even nonlesional skin in patients with atopic dermatitis has been shown to display altered ceramide content and composition compared with healthy skin [40]. Collectively, these reports highlight the necessity of accounting for a wide range of potential confounding factors when analyzing stratum corneum lipids in the clinical setting, in order to ensure accurate interpretation and meaningful application of the findings.

## 4 Application of Stratum Corneum Ceramide Information as Biomarkers in Atopic Dermatitis Management

Currently, noninvasive collection of the stratum corneum by tape‐stripping makes it possible to obtain extensive lipidomic data, which is increasingly being explored as biomarkers in the management of AD. Stratum corneum ceramides in AD lesions differ from those in healthy skin; importantly, studies have shown that after treatment with dupilumab, ceramide profiles in lesional skin shift toward those of healthy controls in parallel with clinical improvement, supporting their role as biomarkers of disease activity [41]. In our clinical study, we focused on ceramide characteristics during remission. We found that the carbon chain lengths of NDS, NS, and NH ceramides at remission predicted subsequent relapse of AD, whereas clinical severity scores and serum thymus and activation‐regulated chemokine (TARC) levels did not [42]. These results indicate that ceramide profiles in remission or nonlesional skin may serve as sensitive indicators of disease status. Although many serum biomarkers, including TARC, have been reported in ad [43], the fact that the skin is the primary site of disease emphasizes the value of direct skin‐based biomarkers. Combining ceramide profiles with systemic markers may allow for more accurate and individualized management of AD.

The phenotypic heterogeneity and multiple disease trajectories of AD pose significant challenges for understanding its pathophysiology. Nonetheless, advances have been made in stratifying patients and identifying distinct endotypes. Stratification approaches have included clinical features and comorbidities [44, 45, 46], serum biomarkers and microbiome analysis [47, 48], and multiomics technologies including single‐cell and spatial transcriptomics [49, 50, 51]. In this context, stratum corneum ceramide profiling offers an additional stratification dimension. For instance, lipidomic analyses at 2 months of age revealed that decreased protein‐bound ceramides, increased unsaturated sphingomyelin species, and increased short‐chain NS‐ and AS‐ceramides are associated with heightened risk of developing ad [52]. Furthermore, AD patients with a history of eczema herpeticum, a severe infectious complication, display distinct stratum corneum lipid profiles compared with those without such history [53]. Thus, ceramide abnormalities not only reflect disease activity but may also predict onset, relapse, and disease trajectory, providing valuable opportunities for individualized management.

It is generally considered that the biosynthesis and metabolism of stratum corneum lipids and circulating lipids are independent processes. In recent years, however, circulating ceramides and sphingolipids have attracted increasing attention as potential disease biomarkers and phenotypic correlates in a variety of disorders. These include bronchial asthma, which, like AD, is characterized by type 2 inflammation [54, 55, 56]. Although abnormalities of stratum corneum ceramides in AD have been extensively investigated, circulating sphingoid lipids in AD have until recently received little attention. We previously reported that several circulating lipid species are altered in AD patients compared with healthy controls, and that a subset of these alterations correlates with the clinical severity of dermatitis [57, 58]. Considering that AD involves not only the skin but also systemic inflammation and multiple comorbidities, it is reasonable to assume that type 2 inflammatory activity may also affect circulating lipid profiles. Indeed, associations between serum lipid alterations and AD comorbidities have been reported [53] These observations emphasize the need for future studies that address not only changes in stratum corneum lipids but also their relationships with systemic lipid metabolism and its dynamic alterations in patients with AD.

## 5 Conclusion: Future Clinical Applications of Stratum Corneum Ceramides

In this review, we have summarized fundamental knowledge regarding stratum corneum ceramides, their abnormalities in patients with AD, and their potential applications in clinical practice. Because stratum corneum ceramides and lipids can be analyzed by simple and noninvasive techniques that provide vast amounts of information, they have attracted considerable attention for their clinical utility [59]. As discussed above, AD is an exceptionally heterogeneous disorder, and stratum corneum ceramides and lipids are subject to diverse influences, including genetic background, environmental exposures, and systemic comorbidities. At the same time, because these lipids constitute the frontline barrier at the interface between the human body and the external environment, they are capable of directly reflecting the current state of the skin while inherently integrating the effects of these multiple factors. Thus, ceramide profiling may serve as a highly useful approach for advancing individualized management of AD in the future. In recent years, the concept of disease modification in AD has been actively discussed [60, 61, 62]. This concept proposes that complete control of inflammation may lead to the achievement of a deep remission that persists long after treatment discontinuation [60]. A critical issue, however, is that the assessment of remission in daily practice is not always straightforward. Our previous findings that stratum corneum ceramide profiles can predict relapse after clinical remission [42] suggest their potential as concrete indicators of deep remission within the framework of disease modification.

Beyond AD and inherited keratinization disorders, the clinical implications of stratum corneum ceramide alterations extend to other diseases. Changes in ceramide composition have been well documented in psoriasis, another major inflammatory skin disease [63], and alterations in stratum corneum ceramide profiles accompanied by impaired barrier function have also been reported in patients with type 2 diabetes [39]. Interestingly, patients with eosinophilic esophagitis—a disorder primarily affecting the esophageal epithelium—have also been reported to exhibit abnormal ceramide profiles in the skin [64]. This observation suggests that skin lipid alterations may mirror systemic epithelial barrier dysfunction, linking cutaneous findings to barrier abnormalities in other organs. Such cross‐organ lipid phenotypes highlight the potential of stratum corneum lipid profiling not only as a marker of skin barrier integrity but also as a window into broader epithelial health.

Although many challenges remain in the clinical application of stratum corneum ceramide and lipid profiling, their noninvasive nature and the ease of sampling are noteworthy advantages. In the future, further clinical development, together with integration of ceramide information into broader multiparametric datasets, is expected to contribute not only to personalized medicine but also to the realization of disease modification in AD.

## Funding

This study was supported by LEO Fondet the 2022 Research Grant of the Japanese Dermatological Association for Basic Medicine (sponsored by Shiseido Co. Ltd.).

## Conflicts of Interest

The author declares no conflicts of interest.

## Data Availability

Data sharing not applicable to this article as no datasets were generated or analysed during the current study.

## References

[1] T. Sakai and Y. Hatano , “Stratum Corneum pH and Ceramides: Key Regulators and Biomarkers of Skin Barrier Function in Atopic Dermatitis,” Journal of Dermatological Science 118, no. 2 (2025): 51–57, 10.1016/j.jdermsci.2025.04.001. 40246650

[2] H. Shimizu , Shimizu's Dermatology, 2nd ed. (John Wiley & Sons, Ltd, 2017).

[3] L. A. Beck , M. J. Cork , M. Amagai , et al., “Type 2 Inflammation Contributes to Skin Barrier Dysfunction in Atopic Dermatitis,” JID Innovations 2, no. 5 (2022): 100131, 10.1016/j.xjidi.2022.100131. 36059592 PMC9428921

[4] A. Kihara , “Synthesis and Degradation Pathways, Functions, and Pathology of Ceramides and Epidermal Acylceramides,” Progress in Lipid Research 63 (2016): 50–69, 10.1016/j.plipres.2016.04.001. 27107674

[5] P. M. Elias and J. S. Wakefield , “Mechanisms of Abnormal Lamellar Body Secretion and the Dysfunctional Skin Barrier in Patients With Atopic Dermatitis,” Journal of Allergy and Clinical Immunology 134, no. 4 (2014): 781–791.e781, 10.1016/j.jaci.2014.05.048. 25131691 PMC4186911

[6] P. M. Elias and Kenneth R. Feingold , Skin Barrier (Taylor & Francis Group, 2006).

[7] P. M. Elias and G. K. Menon , “Structural and Lipid Biochemical Correlates of the Epidermal Permeability Barrier,” Advances in Lipid Research 24 (1991): 1–26, 10.1016/b978-0-12-024924-4.50005-5. Elsevier. 1763710

[8] L. N. Marekov and P. M. Steinert , “Ceramides Are Bound to Structural Proteins of the Human Foreskin Epidermal Cornified Cell Envelope,” Journal of Biological Chemistry 273, no. 28 (1998): 17763–17770, 10.1074/jbc.273.28.17763. 9651377

[9] M. Lundborg , A. Narangifard , C. L. Wennberg , E. Lindahl , B. Daneholt , and L. Norlén , “Human Skin Barrier Structure and Function Analyzed by Cryo‐EM and Molecular Dynamics Simulation,” Journal of Structural Biology 203, no. 2 (2018): 149–161, 10.1016/j.jsb.2018.04.005. 29702212

[10] M. Suzuki , Y. Ohno , and A. Kihara , “Whole Picture of Human Stratum Corneum Ceramides, Including the Chain‐Length Diversity of Long‐Chain Bases,” Journal of Lipid Research 63, no. 7 (2022): 100235, 10.1016/j.jlr.2022.100235. 35654151 PMC9240646

[11] Y. Masukawa , H. Narita , E. Shimizu , et al., “Characterization of Overall Ceramide Species in Human Stratum Corneum,” Journal of Lipid Research 49, no. 7 (2008): 1466–1476, 10.1194/jlr.M800014-JLR200. 18359959

[12] G. Grubauer , P. M. Elias , and K. R. Feingold , “Transepidermal Water Loss: The Signal for Recovery of Barrier Structure and Function,” Journal of Lipid Research 30, no. 3 (1989): 323–333. 2723540

[13] M. Tominaga , S. Ozawa , S. Tengara , H. Ogawa , and K. Takamori , “Intraepidermal Nerve Fibers Increase in Dry Skin of Acetone‐Treated Mice,” Journal of Dermatological Science 48, no. 2 (2007): 103–111, 10.1016/j.jdermsci.2007.06.003. 17643268

[14] T. Okawa , Y. Yamaguchi , S. Takada , et al., “Oral Administration of Collagen Tripeptide Improves Dryness and Pruritus in the Acetone‐Induced Dry Skin Model,” Journal of Dermatological Science 66, no. 2 (2012): 136–143, 10.1016/j.jdermsci.2012.02.004. 22410290

[15] H. Zhai , Y. H. Leow , and H. I. Maibach , “Human Barrier Recovery After Acute Acetone Perturbation: An Irritant Dermatitis Model,” Clinical and Experimental Dermatology 23, no. 1 (1998): 11–13, 10.1046/j.1365-2230.1998.00310.x. 9667101

[16] W. M. Holleran , E. I. Ginns , G. K. Menon , et al., “Consequences of Beta‐Glucocerebrosidase Deficiency in Epidermis. Ultrastructure and Permeability Barrier Alterations in Gaucher Disease,” Journal of Clinical Investigation 93, no. 4 (1994): 1756–1764, 10.1172/jci117160. 8163674 PMC294236

[17] M. Schmuth , M. Q. Man , F. Weber , et al., “Permeability Barrier Disorder in Niemann‐Pick Disease: Sphingomyelin‐Ceramide Processing Required for Normal Barrier Homeostasis,” Journal of Investigative Dermatology 115, no. 3 (2000): 459–466, 10.1046/j.1523-1747.2000.00081.x. 10951284

[18] T. Takeichi and M. Akiyama , “Inherited Ichthyosis: Non‐Syndromic Forms,” Journal of Dermatology 43, no. 3 (2016): 242–251, 10.1111/1346-8138.13243. 26945532

[19] M. A. Aldahmesh , J. Y. Mohamed , H. S. Alkuraya , et al., “Recessive Mutations in ELOVL4 Cause Ichthyosis, Intellectual Disability, and Spastic Quadriplegia,” American Journal of Human Genetics 89, no. 6 (2011): 745–750, 10.1016/j.ajhg.2011.10.011. 22100072 PMC3234380

[20] W. Li , R. Sandhoff , M. Kono , et al., “Depletion of Ceramides With Very Long Chain Fatty Acids Causes Defective Skin Permeability Barrier Function, and Neonatal Lethality in ELOVL4 Deficient Mice,” International Journal of Biological Sciences 3, no. 2 (2007): 120–128, 10.7150/ijbs.3.120. 17311087 PMC1796950

[21] F. Jobard , C. Lefèvre , A. Karaduman , et al., “Lipoxygenase‐3 (ALOXE3) and 12(R)‐lipoxygenase (ALOX12B) are Mutated in Non‐Bullous Congenital Ichthyosiform Erythroderma (NCIE) Linked to Chromosome 17p13.1,” Human Molecular Genetics 11, no. 1 (2002): 107–113, 10.1093/hmg/11.1.107. 11773004

[22] J. L. Moran , H. Qiu , A. Turbe‐Doan , et al., “A Mouse Mutation in the 12R‐Lipoxygenase, Alox12b, Disrupts Formation of the Epidermal Permeability Barrier,” Journal of Investigative Dermatology 127, no. 8 (2007): 1893–1897, 10.1038/sj.jid.5700825. 17429434

[23] P. Krieg , S. Rosenberger , S. de Juanes , et al., “Aloxe3 Knockout Mice Reveal a Function of Epidermal Lipoxygenase‐3 as Hepoxilin Synthase and Its Pivotal Role in Barrier Formation,” Journal of Investigative Dermatology 133, no. 1 (2013): 172–180, 10.1038/jid.2012.250. 22832496

[24] E. Guttman‐Yassky , Y. Renert‐Yuval , and P. M. Brunner , “Atopic Dermatitis,” Lancet 405, no. 10478 (2025): 583–596, 10.1016/s0140-6736(24)02519-4. 39955121

[25] Z. C. Chiesa Fuxench , J. Wan , S. Wang , et al., “Fracture Risk Among Adults With Atopic Dermatitis: A Population‐Based Cohort Study in the United Kingdom,” Journal of the European Academy of Dermatology and Venereology 39, no. 9 (2025): e816–e819, 10.1111/jdv.20728. 40353612

[26] I. M. Mukovozov , D. E. Morra , D. Giustini , M. Tadrous , A. M. Cheung , and A. M. Drucker , “Atopic Dermatitis and Bone Health: A Systematic Review,” Journal of the European Academy of Dermatology and Venereology 35, no. 3 (2021): 615–628, 10.1111/jdv.16895. 32853421

[27] D. Fehr , V. H. Huynh‐Tran , L. Maintz , et al., “Deciphering the Connection Between Atopic Dermatitis and Cardiovascular Diseases: Analysis of Clinical Associations and Cardiometabolic Proteins,” Allergy 80, no. 8 (2025): 2187–2200, 10.1111/all.16588. 40386898

[28] J. Ishikawa , H. Narita , N. Kondo , et al., “Changes in the Ceramide Profile of Atopic Dermatitis Patients,” Journal of Investigative Dermatology 130, no. 10 (2010): 2511–2514, 10.1038/jid.2010.161. 20574438

[29] D. Y. M. Leung , E. Berdyshev , and E. Goleva , “Cutaneous Barrier Dysfunction in Allergic Diseases,” Journal of Allergy and Clinical Immunology 145, no. 6 (2020): 1485–1497, 10.1016/j.jaci.2020.02.021. 32507227 PMC7291847

[30] E. Berdyshev , E. Goleva , I. Bronova , et al., “Lipid Abnormalities in Atopic Skin Are Driven by Type 2 Cytokines,” JCI Insight 3, no. 4 (2018): e98006, 10.1172/jci.insight.98006. 29467325 PMC5916244

[31] Y. Hatano , H. Terashi , S. Arakawa , and K. Katagiri , “Interleukin‐4 Suppresses the Enhancement of Ceramide Synthesis and Cutaneous Permeability Barrier Functions Induced by Tumor Necrosis Factor‐Alpha and Interferon‐Gamma in Human Epidermis,” Journal of Investigative Dermatology 124, no. 4 (2005): 786–792, 10.1111/j.0022-202X.2005.23651.x. 15816837

[32] J. M. Jensen , R. Fölster‐Holst , A. Baranowsky , et al., “Impaired Sphingomyelinase Activity and Epidermal Differentiation in Atopic Dermatitis,” Journal of Investigative Dermatology 122, no. 6 (2004): 1423–1431, 10.1111/j.0022-202X.2004.22621.x. 15175033

[33] E. B. Haddad , S. L. Cyr , K. Arima , R. A. McDonald , N. A. Levit , and F. O. Nestle , “Current and Emerging Strategies to Inhibit Type 2 Inflammation in Atopic Dermatitis,” Dermatologic Therapy (Heidelberg) 12, no. 7 (2022): 1501–1533, 10.1007/s13555-022-00737-7. PMC9276864 35596901

[34] L. Norlén , I. Nicander , B. Lundh Rozell , S. Ollmar , and B. Forslind , “Inter‐ and Intra‐Individual Differences in Human Stratum Corneum Lipid Content Related to Physical Parameters of Skin Barrier Function in Vivo,” Journal of Investigative Dermatology 112, no. 1 (1999): 72–77, 10.1046/j.1523-1747.1999.00481.x. 9886267

[35] P. Jančálková , M. Kopečná , M. Kurka , et al., “Skin Barrier Fine Tuning Through Low‐Temperature Lipid Chain Transition,” Journal of Investigative Dermatology 143, no. 12 (2023): 2427–2435.e2423, 10.1016/j.jid.2023.06.193. 37394058

[36] J. Ishikawa , Y. Shimotoyodome , S. Ito , et al., “Variations in the Ceramide Profile in Different Seasons and Regions of the Body Contribute to Stratum Corneum Functions,” Archives of Dermatological Research 305, no. 2 (2013): 151–162, 10.1007/s00403-012-1286-5. 22987221

[37] N. Yoshikawa , G. Imokawa , K. Akimoto , K. Jin , Y. Higaki , and M. Kawashima , “Regional Analysis of Ceramides Within the Stratum Corneum in Relation to Seasonal Changes,” Dermatology 188, no. 3 (1994): 207–214, 10.1159/000247141. 8186510

[38] H. Emmert , H. Baurecht , F. Thielking , et al., “Stratum Corneum Lipidomics Analysis Reveals Altered Ceramide Profile in Atopic Dermatitis Patients Across Body Sites With Correlated Changes in Skin Microbiome,” Experimental Dermatology 30, no. 10 (2021): 1398–1408, 10.1111/exd.14185. 32885529

[39] J. H. Kim , N. Y. Yoon , D. H. Kim , et al., “Impaired Permeability and Antimicrobial Barriers in Type 2 Diabetes Skin Are Linked to Increased Serum Levels of Advanced Glycation End‐Product,” Experimental Dermatology 27, no. 8 (2018): 815–823, 10.1111/exd.13466. 29151267

[40] J. Kim , B. E. Kim , E. Goleva , et al., “Alterations of Epidermal Lipid Profiles and Skin Microbiome in Children With Atopic Dermatitis,” Allergy, Asthma & Immunology Research 15, no. 2 (2023): 186–200, 10.4168/aair.2023.15.2.186. PMC10079518 37021505

[41] E. Berdyshev , E. Goleva , R. Bissonnette , et al., “Dupilumab Significantly Improves Skin Barrier Function in Patients With Moderate‐To‐Severe Atopic Dermatitis,” Allergy 77, no. 11 (2022): 3388–3397, 10.1111/all.15432. 35815904

[42] Y. Sho , T. Sakai , T. Sato , et al., “Stratum Corneum Ceramide Profiles Provide Reliable Indicators of Remission and Potential Flares in Atopic Dermatitis,” Journal of Investigative Dermatology 142, no. 12 (2022): 3184–3191.e3187, 10.1016/j.jid.2022.06.012. 35870561

[43] Y. Renert‐Yuval , J. P. Thyssen , R. Bissonnette , et al., “Biomarkers in Atopic Dermatitis‐A Review on Behalf of the International Eczema Council,” Journal of Allergy and Clinical Immunology 147, no. 4 (2021): 1174–1190.e1171, 10.1016/j.jaci.2021.01.013. 33516871 PMC11304440

[44] L. Maintz , T. Welchowski , N. Herrmann , et al., “Machine Learning‐Based Deep Phenotyping of Atopic Dermatitis: Severity‐Associated Factors in Adolescent and Adult Patients,” JAMA Dermatology 157, no. 12 (2021): 1414–1424, 10.1001/jamadermatol.2021.3668. 34757407 PMC8581798

[45] L. Maintz , M. T. Schmitz , N. Herrmann , et al., “Atopic Dermatitis: Correlation of Distinct Risk Factors With Age of Onset in Adulthood Compared to Childhood,” Allergy 78, no. 8 (2023): 2181–2201, 10.1111/all.15721. 36946297

[46] I. Kortekaas Krohn , F. M. S. Badloe , N. Herrmann , et al., “Immunoglobulin E Autoantibodies in Atopic Dermatitis Associate With Type‐2 Comorbidities and the Atopic March,” Allergy 78, no. 12 (2023): 3178–3192, 10.1111/all.15822. 37489049

[47] D. S. Bakker , S. Nierkens , E. F. Knol , et al., “Confirmation of Multiple Endotypes in Atopic Dermatitis Based on Serum Biomarkers,” Journal of Allergy and Clinical Immunology 147, no. 1 (2021): 189–198, 10.1016/j.jaci.2020.04.062. 32526312

[48] A. S. L. Tay , C. Li , T. Nandi , et al., “Atopic Dermatitis Microbiomes Stratify Into Ecologic Dermotypes Enabling Microbial Virulence and Disease Severity,” Journal of Allergy and Clinical Immunology 147, no. 4 (2021): 1329–1340, 10.1016/j.jaci.2020.09.031. 33039480

[49] A. Sekita , H. Kawasaki , A. Fukushima‐Nomura , et al., “Multifaceted Analysis of Cross‐Tissue Transcriptomes Reveals Phenotype‐Endotype Associations in Atopic Dermatitis,” Nature Communications 14, no. 1 (2023): 6133, 10.1038/s41467-023-41857-8. PMC10545679 37783685

[50] A. Fukushima‐Nomura , H. Kawasaki , K. Yashiro , et al., “An Unbiased Tissue Transcriptome Analysis Identifies Potential Markers for Skin Phenotypes and Therapeutic Responses in Atopic Dermatitis,” Nature Communications 16, no. 1 (2025): 4981, 10.1038/s41467-025-59340-x. PMC12130345 40456762

[51] Y. Mitamura , M. Reiger , J. Kim , et al., “Spatial Transcriptomics Combined With Single‐Cell RNA‐Sequencing Unravels the Complex Inflammatory Cell Network in Atopic Dermatitis,” Allergy 78, no. 8 (2023): 2215–2231, 10.1111/all.15781. 37312623

[52] E. Berdyshev , J. Kim , B. E. Kim , et al., “Stratum Corneum Lipid and Cytokine Biomarkers at Age 2 Months Predict the Future Onset of Atopic Dermatitis,” Journal of Allergy and Clinical Immunology 151, no. 5 (2023): 1307–1316, 10.1016/j.jaci.2023.02.013. 36828081

[53] E. Berdyshev , E. Goleva , I. Bronova , et al., “Signaling Sphingolipids Are Biomarkers for Atopic Dermatitis Prone to Disseminated Viral Infections,” Journal of Allergy and Clinical Immunology 150, no. 3 (2022): 640–648, 10.1016/j.jaci.2022.02.027. 35304160 PMC9463085

[54] Y. Chen , A. Checa , P. Zhang , et al., “Sphingolipid Classes and the Interrelationship With Pediatric Asthma and Asthma Risk Factors,” Allergy 79, no. 2 (2024): 404–418, 10.1111/all.15942. 38014461 PMC11175620

[55] R. H. Choi , S. M. Tatum , J. D. Symons , S. A. Summers , and W. L. Holland , “Ceramides and Other Sphingolipids as Drivers of Cardiovascular Disease,” Nature Reviews. Cardiology 18, no. 10 (2021): 701–711, 10.1038/s41569-021-00536-1. 33772258 PMC8978615

[56] M. Trayssac , Y. A. Hannun , and L. M. Obeid , “Role of Sphingolipids in Senescence: Implication in Aging and Age‐Related Diseases,” Journal of Clinical Investigation 128, no. 7 (2018): 2702–2712, 10.1172/jci97949. 30108193 PMC6025964

[57] T. Sakai , N. Herrmann , L. Maintz , et al., “Serum Sphingosine‐1‐Phosphate Is Elevated in Atopic Dermatitis and Associated With Severity,” Allergy 76, no. 8 (2021): 2592–2595, 10.1111/all.14826. 33764548

[58] T. Sakai , N. Herrmann , L. Maintz , et al., “Altered Serum Phospholipids in Atopic Dermatitis and Association With Clinical Status,” JID Innovations 2, no. 2 (2022): 100092, 10.1016/j.xjidi.2021.100092. 35199091 PMC8844610

[59] R. Sanabria‐de la Torre , T. Montero‐Vílchez , J. García‐Gavín , and S. Arias‐Santiago , “Current Insights on Lipidomics in Dermatology: A Systematic Review,” Journal of Investigative Dermatology 145, no. 5 (2025): 1105–1116.e1106, 10.1016/j.jid.2024.09.003. 39303909

[60] T. Bieber , L. Maintz , G. E. Phad , and M. C. Brüggen , “From Disease Control to Disease Modification: The Atopic Dermatitis Disease Activity Index,” Allergy (2025), 10.1111/all.70036. PMC12862550 40879255

[61] T. Bieber , “Disease Modification in Inflammatory Skin Disorders: Opportunities and Challenges,” Nature Reviews. Drug Discovery 22, no. 8 (2023): 662–680, 10.1038/s41573-023-00735-0. 37443275

[62] J. I. Silverberg , M. Gooderham , N. Katoh , et al., “Combining Treat‐To‐Target Principles and Shared Decision‐Making: International Expert Consensus‐Based Recommendations With a Novel Concept for Minimal Disease Activity Criteria in Atopic Dermatitis,” Journal of the European Academy of Dermatology and Venereology 38, no. 11 (2024): 2139–2148, 10.1111/jdv.20229. 38989857

[63] J. Rousel , C. Mergen , M. E. Bergmans , et al., “Lesional Psoriasis Is Associated With Alterations in the Stratum Corneum Ceramide Profile and Concomitant Decreases in Barrier Function,” Experimental Dermatology 33, no. 10 (2024): e15185, 10.1111/exd.15185. 39382258

[64] E. Goleva , D. Y. Leung , K. Chaiboonma , et al., “Cutaneous Ceramide Synthesis Is Dysregulated in Pediatric Eosinophilic Esophagitis,” Journal of Allergy and Clinical Immunology 155, no. 6 (2025): 2075–2079.e2071, 10.1016/j.jaci.2025.02.024. 40020933

